# Association Between Glaucoma and Mental Health Disorders Based on a Large National Database

**DOI:** 10.18502/jovr.v20.17535

**Published:** 2025-12-08

**Authors:** Mohammad Delsoz, Hina Raja, Zain S. Hussain, Vahid Mohammad Zadeh, Muhammad Elahi, Jesse Wesberry, Brian Jerkins, Claire Wright, Elliott Kanner, Siamak Yousefi

**Affiliations:** ^1^Hamilton Eye Institute, Department of Ophthalmology, University of Tennessee Health Science Center, Memphis, TN, USA; ^2^Dean McGee Eye Institute, Oklahoma City, OK, USA; ^3^University of Medicine and Health Sciences, Basseterre, Saint Kitts and Nevis; ^4^David Geffen School of Medicine, University of California Los Angeles, Los Angeles, USA; ^5^Quillen College of Medicine, East Tennessee State University, TN, USA; ^6^Bascom Palmer Eye Institute, Department of Ophthalmology, University of Miami, Miami, FL, USA; ^7^Department of Electrical and Computer Engineering, University of Miami, Miami, FL, USA

**Keywords:** Anxiety, Bipolar, Glaucoma, Major Depressive Disorder, Schizophrenia

## Abstract

**Purpose:**

To investigate the association between glaucoma and various mental health disorders and to examine whether there were indications of effect measure modification of this association in Black compared to Non-Black populations.

**Methods:**

The study included 65,140 individuals from the Medical Expenditure Panel Survey database, with an in-depth focus on 15,016 patients suffering from glaucoma or specific mental health disorders. We included patients aged 18 and above diagnosed with glaucoma or specific mental health disorders based on International Classification of Diseases codes from 2017 to 2020.

**Results:**

Out of the 65,140 patients, 1492, 6359, 5756, 786, and 209 were diagnosed with glaucoma, anxiety, major depressive disorder (MDD), bipolar disorder, and schizophrenia, respectively. Of the 1492 glaucoma patients, 196 (13.2%) were diagnosed with anxiety, 183 (12.2%) with MDD, 20 (1.3%) with bipolar, and 15 (1%) with schizophrenia. The unadjusted OR (95% CI) was calculated for the association between glaucoma and anxiety (OR = 1.36 [1.17-1.58]) *P* < 0.001), glaucoma and MDD (OR = 1.4 [1.20-164], *P* < 0.001), glaucoma and bipolar (OR = 1.08 [0.69-1.6], *P* = 0.71), and glaucoma and schizophrenia and (OR = 3.08 [1.82-5.21], *P* < 0.001). After adjustment for confounding factors, the association between glaucoma and anxiety, glaucoma and MDD, and glaucoma and schizophrenia remained statistically significant. Furthermore, for this association after interaction analysis, the interaction term of glaucoma and race with MDD (OR = 1.02 [0.6-1.5], *P* = 0.9), anxiety (OR = 0.64 [0.39-1], *P* = 0.069), and schizophrenia (OR = 2.8 [0.9-8.6], *P* = 0.9) didn't yield a significant evidence of effect measure modifications in Black compared to Non-Black groups after Bonferroni correction.

**Conclusion:**

There was a statistically significant association between glaucoma and MDD, anxiety, and schizophrenia. However, for this association, there was no significant evidence of effect measure modifications in Black compared to Non-Black populations.

##  INTRODUCTION

Glaucoma is a diverse set of illnesses characterized by cupping of the optic nerve head and impairment of the visual field, and is the most common cause of irreversible blindness worldwide.^[1,2]^ Even after receiving appropriate therapy, which generally consists of laser, topical eye drops, and surgery, about 10% of patients with glaucoma experience vision loss.^[3]^ More than 120,000 people in the United States are blind due to glaucoma, making up between 9% and 12% of all cases of blindness.^[3]^ Compared to the general population, people with glaucoma may be more likely to suffer from mental health disorders, possibly due to their heightened awareness of the possibility of permanent visual loss.^[4,5]^ Several risk factors for glaucoma have been identified, including advanced age, family history, race or ethnicity, myopia, and obstructive sleep apnea.^[6,7,8,9,10,11,12]^ Elevated intraocular pressure (IOP), however, is the major modifiable risk factor, as increased IOP can lead to optic nerve damage.^[7]^

**Recently, the effect of eye diseases on psychological disorders has been investigated, and associations have been identified.^[13]^ Epidemiological research has found that about 50.6% of all patients with mental health disorders also suffer from another chronic health issue.^[14]^ Individuals with mental health disorders may have a higher risk for developing ocular disease due to the presence of certain risk factors, including cigarette smoking,^[15]^ metabolic syndrome and diabetes,^[16]^ and the use of antipsychotic medications, which predispose them to both cataract^[17]^ and dry eye.^[18]^**

Several studies have compared the rates of mental health disorders, particularly anxiety and depression, in patients with glaucoma versus subjects without glaucoma, and have reported inconsistent correlations.^[13,19,20,21]^ It is known that the prevalence of mental health disorders in patients with glaucoma also varies greatly across regions.^[3,22,23,24]^ Identifying an association between mental health disorders and glaucoma, and whether the significance of this association varies across different racial and ethnic groups, may lead to a more comprehensive and effective treatment plan for patients from that ethnic group.

The purpose of this study is to assess the association between glaucoma and selected mental health disorders and to examine whether race and ethnicity influence this association using a statistical phenomenon called effect measure modification (EMM)^[25]^ based on a large national database to gain some insight into glaucoma as a complex disease.

##  METHODS 

We used the Medical Expenditure Panel Survey (MEPS) dataset to investigate the association between glaucoma and mental health disorders. MEPS is a national survey of the civilian, noninstitutionalized population of the United States conducted annually by the Agency for Healthcare Research and Quality (AHRQ).^[26]^ MEPS gathers comprehensive data on each household member's demographics, health conditions, health status, usage of medical services, costs and payment sources, accessibility to care, satisfaction with care, health insurance coverage, earnings, and employment. We used the full-year consolidated data file and the medical condition file from MEPS. The MEPS Medical Provider Component has been reviewed and approved by the RTI International IRB, established under a multi-project assurance (Federal Assurance Number 3331) granted by the Office for Protection from Research Risks (OPRR). The institutional review board (IRB) has been approved by the University of Tennessee Health Science Center (IRB Number: 20-07817-XP) based on the Westat IRB and the Office for Protection from Research Risks. This study adhered to the tenets of the Helsinki Declaration.

To conduct this retrospective analysis, we gathered data from January 1, 2017, through December 31, 2020. Patients above the age of 18 and under the age of 85 were included.

The diagnosis of both glaucoma and mental health disorders in the MEPS database was based on self-reports of prior provider-diagnosed conditions, which were then converted into ICD codes by researchers for database construction. Therefore, the following ICD codes, such as for glaucoma (ICD-10, code H40), Major Depressive Disorder (MDD; ICD-10, code F32), anxiety (ICD-10, code F41), schizophrenia (ICD-10, code F20), and bipolar (ICD-10, code F31) were used to include patients. Patients with multiple diagnosis codes for the same ailment were counted only once.

Socioeconomic and demographic characteristics were assessed, including age, gender, race/ethnicity, marital status, educational level, income, and region. In addition, annual hospital visits and systemic medical conditions were incorporated using the Charlson Comorbidity Index (CCI).

**Table 1:** Baseline demographic and clinical characteristics of glaucoma patients with any of the four mental health disorders and glaucoma patients without any of the mental disorders

**Characteristics**	**Glaucoma with any of mental disorder (** ***n*** ** = 317 patients)**	**Glaucoma without mental disorder (** ***n*** ** = 1175 patients)**	***P*** **-value**
Mean age (SD), year	68.3 (12.2)	70.2 (12)	0.45
Gender (%)			< 0.001
Male	100 (31.5%)	526 (44.8%)	
Female	217 (68.5%)	649 (55.2%)	
Race/Ethnicity (%)			< 0.001
White	253 (79.8%)	774 (65.9%)	
Black	46 (14.5%%)	310 (26.4%)	
Amer Indian/Alaska national/ Asian/Native Hawaiian	9 (2.8%)	68 (5.8%)	
Unknown	9 (2.8%)	23 (2.0%)	
Region (%)			< 0.001
West	301 (20.2%)	16206 (25.4%)	
South	554 (37%)	24116 (37.7%)	
Midwest	324 (21.7%)	12940 (20.2%)	
Northeast	313 (21%)	10644 (16.6%)	
Marital status (%)			< 0.001
married	118 (37.2%)	618 (52.6%)	
widowed	69 (21.8%)	251 (21.4%)	
divorced	66 (20.8%)	169 (14.4%)	
Separated	18 (5.7%)	24 (2%)	
Never married (single)	46 (14.5%)	113 (9.6%)	
Highest Degree (%)			0.24
No degree	93 (29.3%)	301 (25.6%)	
GED	13 (4.1%)	35 (3.0%)	
High school diploma	126 (39.7%)	472 (40.2%)	
Bachelor's degree	53 (16.7%)	190 (16.2%)	
Master's degree	25 (7.9%)	138 (11.7%)	
Doctorate degree	7 (2.2%)	39 (3.3%)	
Family income (%)			< 0.001
< 30000	143 (45.1%)	379 (32.3%)	
30000-89999	110 (34.7%)	488 (41.5%)	
90000-149999	47 (14.8%)	195 (16.6%)	
150000-209999	12 (3.8%)	65 (5.5%)	
> 210000	5 (1.6%)	48 (4.1%)	
Annual visit of OPD (Mean), SD	1.58 (5.99)	1.37 (5.37)	0.54
Annual visit of emergency dept (Mean), SD	0.40 (1)	0.27 (0.69)	0.007
CCI score			< 0.001
0	106 (33.46%)	412 (35.12%)	
1-2	113 (35.67%)	437 (37.2%)	
3-4	51 (16.34%)	180 (15.25%)	
≥ 5	47 (14.53%)	145 (12.4%)	
SD, standard deviation; GED, general education; OPD, outpatient department; CCI, Charlson Comorbidity Index Score.			

**Table 2:** Prevalence of each of the mental disorders in non-glaucoma and glaucoma patients

**Mental disorders**	**Non-glaucoma (** ***N*** ** = 63648)**		**Glaucoma (** ***N*** ** = 1492)**	
Number/percentage	*N*	%	*N*	%
Anxiety	6359	10%	196	13.2%
MDD	5756	9%	183	12.2%
Bipolar disorder	786	1.2%	20	1.3%
Schizophrenia	209	0.3%	15	1%
MDD, major depressive disorder.				

**Table 3:** Unadjusted and adjusted odds ratios (aOR) of glaucoma with each of the mental disorders

**Mental disorders**	**Glaucoma**			
	**Unadjusted ORs (95% CI)**	***P*** **-value**	**Adjusted ORs (95% CI)**	***P*** **-value**
Anxiety	1.36 (1.17 to 1.58)	< 0.001	1.23 (1.10 to 1.54)	< 0.001
MDD	1.4 (1.20 to 164)	< 0.001	1.2 (1.03 to 1.42)	0.024
Bipolar disorder	1.08 (0.69 to 1.6)	0.71	1.38 (0.88 to 2.18)	0.15
Schizophrenia	3.08 (1.82 to 5.21)	< 0.001	2.89 (1.65 to 5.05)	< 0.001
${}^{*}$ Adjusted with age, gender, race, region, marital status, family income, CCI score, and educational level. MDD, major depressive disorder.				

**Table 4:** Racial differences in the prevalence of mental health disorders among patients with glaucoma and two-way interaction analysis

**Glaucoma**	**Black**	**Non-Black**	***P*** **-value**	
***N*** ** = 1492**	***N*** ** = 356**	***N*** ** = 1136**		
Racial differences among patients with glaucoma				
Anxiety %	5.9%	16.13%	< 0.001	
MDD %	8.9%	14.16%	0.038	
Schizophrenia %	2.8%	0.46%	< 0.001	
Bipolar %	0.8%	1.5%	0.5	
Interaction effects and Bonferroni corrections				
Mental health disease Predictors		*P*-value	OR (95% CI)	*P*-values ${}^{*}$
MDD	Glaucoma Race Main effect (interaction)	< 0.001 < 0.001 0.9	1.46 (1.25 to 1.7) 1.68 (1.4 to 1.89) 1.02 (0.6 to 1.5)	< 0.001 < 0.001 0.225
Anxiety	Glaucoma Race Main effect (interaction)	< 0.001 < 0.001 0.069	1.42 (1.2 to 1.66) 1.68 (1.49 to 1.9) 0.64 (0.39 to 1)	< 0.001 < 0.001 0.0172
Schizophrenia.	Glaucoma Race Main effect (interaction)	< 0.001 < 0.001 0.9	3.08 (1.8 to 5.2) 1.6 (1.4 to 1.8) 2.8 (0.9 to 8.6)	< 0.001 < 0.001 0.225
Bipolar	Glaucoma Race Main effect (interaction)	0.7 < 0.001 0.26	1.1 (0.69 to 1.69) 1.7 (1.5 to 1.9) 0.5 (0.14 to 1.7)	0.175 < 0.001 0.065
${}^{*}$ P-values for testing the interaction effects (Bonferroni-corrected significant threshold = 0.0125). MDD, major depressive disorder.				

### Statistical Analysis 

Descriptive statistics were assessed with mean and standard deviation (SD). For categorical variables, a chi-squared test was used; for continuous variables, an independent *t*-test was used. Odds ratios (ORs) for associations of glaucoma and specified mental health disorders were calculated using the chi-squared test. We adjusted for age, gender, race, marital status, educational level, family income, CCI score, and region using multivariable logistic regression models and computed 95% confidence intervals for calculating the adjusted OR.

Furthermore, to examine EMM between glaucoma and race, a comprehensive multivariable logistic regression model (two-way interaction) was developed to evaluate the effects of glaucoma and race on selected mental health diseases and accounted for multiple measurements based on Bonferroni correction. To account for the complex study design, including stratification, appropriately weighted data and clustering variables from MEPS were applied in all statistical analyses. We conducted the statistical analyses using SPSS (version 29, IBM Corp., Armonk, NY, USA). The alpha level (type I error) of 0.05 was considered statistically significant.

##  RESULTS

A total of 65,140 patients were included in this study. The number of female patients was 34,668 (53.2%). Among these patients, 1492 (2.3%) were diagnosed with glaucoma, 1175 (1.8%) were diagnosed with glaucoma without any of these mental health disorders, and 317 (0.5%) were diagnosed with both glaucoma and any of the aforementioned mental health disorders. The most frequent mental health disorder was anxiety (*N* = 6555: 10%), followed by MDD (*N* = 5939: 9.1%), bipolar disorder (*N* = 806: 1.2%), schizophrenia (*N* = 224: 0.3%), and 3070 (4.7%) patients suffered from more than one mental health disorder.

Table 1 shows the demographics and clinical characteristics of glaucoma with any of the specified mental health disorders and glaucoma without any of the specified mental health disorders. Patients diagnosed with both glaucoma with any of specified mental health disorders were most commonly women (68.5%, *P* < 0.001), of White ethnicity (79.8%, *P* < 0.001), from low annual family income < $30000 (45.1%, *P* < 0.001), and had high mean annual visit of emergency department (0.40 ± 1, *P* < 0.007) for any reason. The highest proportions were female, 217 (68.5%), and had a CCI score of 1-2 (113 [35.67%]).

Table 2 shows the prevalence of mental health disorders (MDD, anxiety, bipolar disorder, and schizophrenia) among patients with and without glaucoma. Of the 63,648 non-glaucomatous patients, 6359 (10%) were diagnosed with anxiety, followed by 5756 (9%) with MDD, 786 (1.2%) with bipolar disorder, and 209 (0.3%) with schizophrenia. Of the 1492 glaucoma patients, 196 (13.2%) were also diagnosed with anxiety, followed by 183 (12.2%) with MDD, 20 (1.3%) with bipolar disorder, and 15 (1%) with schizophrenia.

Table 3 displays the four different types of mental health disorders analyzed: anxiety, MDD, bipolar disorder, and schizophrenia. Among these four diagnoses, we observed a statistically significant association between glaucoma and each of these mental health disorders except bipolar disorder (*P* = 0.15). The adjusted OR (95% CI) for the association between glaucoma and anxiety (aOR = 1.23 [1.10-1.54], *P* < 0.001), glaucoma and MDD (aOR = 1.2 [1.03-1.42], *P*=0.024), glaucoma and schizophrenia (aOR = 2.89 [1.65-5.05], *P* < 0.001) remained statically significant after adjusting for age, sex, race (and/or ethnicity), regions, marital status, family income, CCI scores, and educational levels.

Table 4 demonstrates the ethnic differences in prevalence of the selected mental health disorders in glaucoma patients and the interaction term between glaucoma and race on specific mental health disorders, including MDD, anxiety, and schizophrenia. Black individuals had prevalences of 8.9% (*P* = 0.038), 2.8% (*P* < 0.001), 5.9% (*P* < 0.001), and 1.5% (*P* = 0.5) for MDD, schizophrenia, anxiety, and bipolar disorder, respectively. However, Non-Black individuals had prevalences of 16.13% (*P* < 0.001), 0.46% (*P* < 0.001), 16.3% (*P* < 0.001), and 0.8% (*P* = 0.5) for MDD, schizophrenia, anxiety, and bipolar disorder, respectively. The main effect “glaucoma and race interacts” had an odds ratio of (OR = 1.02 [0.6-1.5], *P* = 0.09), (OR = 0.64 [0.39-1], *P* = 0.9), and (OR = 2.8 [0.9-8.6], *P* = 0.9) with MDD, anxiety, and schizophrenia, respectively (a significant *P*-value was considered as 0.0125, after Bonferroni correction). All proportions in the results section have been adjusted using weighted data.

##  DISCUSSION

We retrospectively investigated the MEPS dataset from January 1, 2017, through December 31, 2020, to identify the association between glaucoma and several mental health disorders. We observed that glaucoma is associated with anxiety, MDD, and schizophrenia, after adjusting for numerous socioeconomic and demographic characteristics, including age, sex, ethnicity, regions, marital status, family income, CCI score, and educational levels. In addition, we further investigated the association of glaucoma and selected mental health disorders based on the race of Black and Non-Black populations.

Overall, anxiety was common among patients with glaucoma in this cohort, with a prevalence of 13.2%, which is substantially lower than the rates reported in Singapore (64%),^[22]^ India (25.%),^[23]^ and China (22.92%);^[27]^ is comparable to published rates in Japan (13.0%)^[21]^ and Turkey(14.0%);^[28]^ and is higher than the rate reported in Germany (5.3%).^[24]^ The prevalence of anxiety in this study was lower than in a previous study, which reported an anxiety prevalence of 17.1% in the US.^[19]^ Prevalence of MDD in this group of patients who were diagnosed with glaucoma was 12.2%, which is comparable to reported rates in Japan (10.9%)^[21]^ and China (16.40%),^[27]^ lower than the published rates in Turkey (57%),^[28]^ India (35.81%),^[23]^ and Singapore (30%),^[22]^ but higher than reported rates in Germany (6.6%).^[24]^ The prevalence of bipolar disorder among patients with glaucoma was 1.3%, followed by 1.0% schizophrenia. Understanding the rates of mental health disorders among adults with glaucoma in different countries is crucial for offering comprehensive patient care. However, it is important to delve into the underlying factors that might contribute to these observed patterns. One possible explanation could be the psychosocial impacts of living with a chronic eye condition like glaucoma. The progressive nature of the disease, potential loss of vision, and the accompanying challenges in daily life might lead to increased stress, anxiety, and depressive symptoms.^[4,5][29]^ Moreover, the limitations imposed by glaucoma on activities that are essential for mental well-being, such as reading or engaging in visual arts, could further exacerbate mental health issues.^[29]^ Additionally, variations in healthcare infrastructure and access to mental health services across different countries could also play a significant role.^[30]^ Disparities in access to quality mental healthcare may result in underdiagnosis or inadequate treatment for individuals with glaucoma, potentially contributing to higher rates of mental health disorders. By exploring these underlying factors, we can gain deeper insights into the intricate relationship between glaucoma and mental health and, in turn, provide more effective interventions and support systems for affected individuals worldwide.

The total prevalence of anxiety in this cohort was 10%, followed by MDD (9.1%), bipolar disorder (1.3%), and schizophrenia (0.3%). The prevalence of glaucoma in this cohort was 2.2% which is comparable with the previously published prevalence of glaucoma worldwide (2.4%).^[31]^

The findings of this investigation demonstrated a significant association between glaucoma and anxiety disorder, MDD, and schizophrenia. The association between glaucoma and bipolar disorder, however, was not statistically significant. Another study found an association between glaucoma and anxiety and depression,^[19]^ while some other studies have not confirmed these findings.^[20,24]^ Our results are consistent with several prior studies that found that glaucoma is associated with mental health disorders.^[19,22,32]^ Glaucoma and being categorized as Non-Black play notable roles in influencing the odds of experiencing various mental health disorders. Our study found a higher prevalence of experiencing mental health disorders, including MDD and anxiety (14.16% and 16.13%, respectively), in Non-Black glaucoma patients compared with Black patients with glaucoma (8.9% and 5.9%, respectively). Our study aligns with previous studies which demonstrated that the Non-Black population in the United States shows a higher lifetime prevalence of MDD compared to the Black population,^[33]^ and they also experience higher rates of generalized anxiety disorder.^[34]^ Researchers attribute these gaps to factors such as protective social networks and strong community ties in Black communities, variations in cultural expressions of distress, and measurement differences.^[33,34]^ When Black populations do develop MDD, it tends to be more persistent and severe, often going untreated due to systemic barriers and limited access to high-quality care.^[35]^ Meanwhile, the Non-Black population may face greater comorbidity with mood disorders and appear more likely to develop anxiety disorders later in life—potentially due to ongoing job stress or late-life relocations—whereas Black groups face early-life stressors that may contribute to a higher risk of post-traumatic stress disorders.^[34]^ These differences underscore the importance of the social environment, cultural coping mechanisms, and healthcare disparities in explaining differences in prevalence rates. In contrast, in the case of schizophrenia, Black patients with glaucoma had a higher prevalence compared to Non-Black patients (2.8% vs. 0.46%). Similarly, recent studies demonstrated that Black patients had a higher prevalence compared to Non-Black patients.^[36,37]^ This disparity is not necessarily indicative of an actual difference in prevalence but may be influenced by factors such as diagnostic biases and systemic inequalities.^[38]^ Clinicians may misinterpret cultural expressions or behaviors, leading to overdiagnosis of schizophrenia in Black individuals, or this disparity may stem from Black patients with schizophrenia more frequently utilizing office-based outpatient treatment.^[38]^ Alternatively, it could be due to Black patients seeking mental health outpatient care receiving schizophrenia diagnoses more often than White patients, possibly because of factors like misdiagnosis of bipolar and other mood disorders.^[36]^

However, the prevalence of bipolar disorder showed no significant difference between the two groups (0.8% vs. 1.5%). Both glaucoma and race independently increase the likelihood of mental health conditions. However, the study reveals that the interaction term between glaucoma and race and ethnicity is not significant. Therefore, for this association, there is no significant evidence of EMM in Black individuals compared to Non-Black individuals. These findings not only support the association of glaucoma and selected mental health disorders but also indicate the absence of racial disparities in the association between glaucoma and selected mental health disorders, including MDD, anxiety, and schizophrenia. Recognizing both medical and broader sociocultural factors is crucial to addressing these disparities, underscoring the need for comprehensive solutions.

The association between glaucoma and mental health disorders such as anxiety disorder, MDD, and schizophrenia is significant for several reasons. Both glaucoma and these mental health disorders have been associated with abnormalities in specific biological pathways, such as the dopaminergic system and the immune system.^[39,40,41,42]^ Understanding these shared pathways could lead to the development of new treatments for both glaucoma and mental health disorders. While schizophrenia (dopaminergic) and mood disorders (serotonergic/noradrenergic) involve distinct pathways, shared mechanisms such as immune dysregulation, oxidative stress, and psychosocial stressors (e.g., chronic disease burden) may link these conditions to glaucoma.^[43,44]^ For example, immune-inflammatory pathways have been implicated in both glaucoma progression and psychiatric disorders. Moreover, antipsychotics prescribed for schizophrenia/bipolar disorder are known to increase glaucoma risk due to increasing intraocular pressure,^[44]^ underscoring clinical relevance. In addition, vision loss, anxiety, and depression can not only impair a person's quality of life^[23]^ but also can negatively affect a person's adherence to treatment, so addressing these conditions together may lead to improved quality of life, therapy adherence, and overall visual outcomes. Finally, glaucoma and mental health disorders share multiple risk factors such as age,^[45,46]^, and genetics^[47,48]^ that highlight both disease complexity and the importance of a multidisciplinary approach to patient care.

Public health awareness and clinician knowledge play crucial roles in improving outcomes and promoting the well-being of individuals affected by glaucoma and mental health disorders. Public health initiatives, including educational campaigns, media outreach, and community events, can enhance awareness and understanding, leading to early detection, reduced stigma, improved access to care, and the adoption of preventive measures and self-care practices. Clinician awareness is also critical for identifying signs and risk factors, regularly screening patients, and providing appropriate treatment and support. Clinicians should be mindful of the connection between glaucoma and mental health disorders, as well as the potential impact of mental health on treatment adherence. Through regular screening, early detection, effective treatment, and interdisciplinary collaboration, individuals can receive the care and support they need.

Our study still has some limitations to consider. First, self-reporting of glaucoma is a major limitation in this study. It may be that a patient who self-reports a mental health disorder is more likely to also self-report a glaucoma diagnosis by virtue of higher health literacy and recall bias. Second, the use of ICD codes (ICD-10) to identify study groups may result in misclassification bias, as some individuals with the condition of interest may not have been correctly identified. Third, the lack of adjustment of glaucoma severity is another limitation. There is a possibility that a specific ethnicity with advanced glaucoma may show a higher rate of mental health disorders. Fourth, the small sample size across racial categories for evidence of effect measure modification. Therefore, future research should focus on larger samples or pooled datasets to enable more detailed, race- or ethnicity-stratified analyses. Furthermore, the study's retrospective nature makes it impossible to establish a temporal relationship between glaucoma and the development of these mental health disorders; this limits the scope of our research to the association between these conditions, as opposed to demonstrating causation. Finally, in addition to diagnostic bias, other sources of bias, including implicit bias and differences in access to ophthalmology and psychiatric care, may also impact findings.

Overall, mental health disorders and glaucoma can have significant consequences on a person's health, including quality of life, treatment adherence difficulties, social isolation, increased healthcare utilization, higher risk of comorbidities, and decreased life expectancy. Therefore, a comprehensive approach to patient care that addresses racial disparities and physical and psychological factors is crucial for improving outcomes and enhancing the overall health and well-being of affected individuals. This may involve coordination between ophthalmologists, mental health professionals, and primary care physicians, as well as support from family and community resources^[49]^ such as American Foundation for Blindness (AFB), Glaucoma Foundation, American Council of the Blind, Prevent Blindness America, Lions Club International, National Federation of the Blind (NFB), and Lighthouse Guild.

In summary, our study showed that there was a statistically significant association between glaucoma and select mental health disorders, namely anxiety, MDD, and schizophrenia. However, there was no association between glaucoma and bipolar disorder. Furthermore, for this association, there was no significant evidence of effect measure modification between Black and Non-Black populations. The consequences of these comorbidities can be severe, impacting the individual's quality of life, functional status, and overall health outcomes. Therefore, clinicians need to be aware of this association, regularly screen their patients, and offer appropriate treatment and support. Further research is needed to better understand the mechanisms underlying the association between glaucoma and mental health disorders and to identify effective interventions to improve patient outcomes.

##  Financial Support and Sponsorship

This work was supported by NIH Grants R01EY033005 (SY), R21EY031725 (SY), and a Challenge Grant from Research to Prevent Blindness (RPB), New York (SY). The funders had no role in study design, data collection and analysis, decision to publish, or preparation of the manuscript.

##  Conflicts of Interest

None.
